# Greening the Vietnamese supply chain: The influence of green logistics knowledge and intellectual capital

**DOI:** 10.1016/j.heliyon.2023.e15953

**Published:** 2023-04-29

**Authors:** Hien Van Vo, Nguyen Phong Nguyen

**Affiliations:** School of Accounting, University of Economics Ho Chi Minh City, Ho Chi Minh City, Viet Nam

**Keywords:** Green logistics, Green knowledge, Green management practices, Environmental performance, Green intellectual capital, Vietnam

## Abstract

Green logistics has recently received considerable attention because of its practical benefits to businesses and the environment, particularly in developing countries. However, there is a lack of empirical evidence regarding the motivations behind green logistics practices and environmental performance. This study explores the impact of green logistics knowledge exploitation on green logistics management practices and green logistics performance. In addition, this study explains the moderating role of green intellectual capital in the relationship between green logistics knowledge exploitation and green logistics management practices. Based on responses from 142 Vietnamese logistics business managers, the data were analyzed using partial least squares structural equation modeling. To our knowledge, this is the first study to confirm 1) the influence of green logistics knowledge exploitation on green logistics performance through the mediating role of green logistics management practices, and 2) the moderating effect of green intellectual capital on the relationship between green logistics knowledge exploitation and green logistics management practices. These results have implications for environmental management practices in logistics operations and could help promote green logistics practices through green logistics knowledge exploitation and its subsequent enhancement of green logistics performance.

## 1 Introduction

Economic development and environmental protection are the dual goals of every country. Enterprises’ environmental protection activities are always highly appreciated as stakeholders are gradually paying more attention to the environment than financial performance [[1], [2], [3], [4], [5]]. Green actions can help businesses expand their markets and increase competitiveness [6]. However, economic growth is always associated with an increase in the consumption of goods and services. The transport, storage, preservation, and consumption of large volumes of goods have caused environmental problems, such as exploitation, excessive use of natural resources and non-renewable materials, recycling, and carbon dioxide (CO^2^) emissions, which are the main causes of climate change and global warming [7]. These consequences are closely related to the nature of the environmentally unfriendly activities of logistics firms [8,9]. Meanwhile, under intense stakeholder pressure, firms must prove their environmental commitment through actions that promote a green environment [2]. Logistics firms are no exception and must pursue appropriate green policies and strategies, such as green logistics practices.

Green logistics is considered an integral part of the green supply chain [8,10,11], and refers to strategies and practices in supply chain management that aim to minimize the negative impact related to the distribution of goods on the ecological environment. This method requires businesses to demonstrate environmental responsibility by focusing on waste management, material recycling, packaging, and transportation [12]. From the sustainable development perspective, green logistics is defined as "producing and distributing goods in a sustainable way, taking account of environmental and social factors" [13] (p. 159). Furthermore, green logistics can provide numerous ecological benefits that exceed customer expectations, creating a contagion effect in the supply chain by raising the environmental awareness of suppliers and customers [14]. Several empirical studies have measured the influence of green logistics practices on environmental and social performance. However, this influence has only been tested extensively in China [15], Australia [16], Ghana [8,9], and South Africa [17], indicating a gap in empirical evidence from other regions.

The researchers of the current study selected Vietnam as the study site because, similar to other developing countries, it has great potential for developing green logistics [18]; these countries are also negatively affected by climate change [6,19]. Therefore, green logistics practices are an appropriate solution to support the sustainable development goals (SDGs) pursued by the Vietnamese government. However, most logistics enterprises in Vietnam are small, their scope of activities is fragmented, and their resources are insufficient, leading to low competitiveness in the logistics sector [20]. In this context, green logistics can be a helpful solution to improve the competitiveness of logistics firms in emerging economies [6,21].

In response to the growing environmental issues faced by logistics firms in emerging markets and the potential competitive benefits of green logistics, the researchers designed a moderated mediation model incorporating green logistics knowledge exploitation (GLKE), green logistics management practices (GLMP), green intellectual capital (GIC), and green logistics performance (GLP). First, the researchers examined the effects of GLKE on GLMP. Most Vietnamese logistics firms operate in traditional ways, focusing on purchasing, distribution, maintenance, and inventory management, but overlook marketing activities, new product development, finance, and customer service [20]. Switching to green logistics requires firms to explore, learn, and receive related knowledge from stakeholders. Subsequently, logistics enterprises transform and apply these to environmental management activities (such as green training, green transport, green energy, green information processing, and distribution). Next, the researchers verified the relationship between GLMP and GLP, which is crucial for Vietnamese logistics firms because this relationship can provide managers with the environmental performance implications of green management (such as reducing energy consumption, CO^2^ emissions, and the costs of environmental compliance, improving the green brand value and the environmental ethics of employees). Based on the relationship between GLKE and GLMP, and GLMP and GLP, the researchers considered the mediating role of GLMP. This is also consistent with studies on green practices; the acquisition/learning of environmental knowledge can impact environmental performance through an organization’s green behaviors [22]. Additionally, the moderating role of intellectual capital, as well as GIC, has also been examined in manufacturing enterprises [23,24]. In a novel undertaking, the current study used GIC as a moderator in logistics activities. As human resources in Vietnamese logistics enterprises are lacking or weak [20], this provides a necessary resource for the transition to green logistics [21]. The researchers hypothesize that as businesses invest more in GIC, GLKE can be more effective in promoting GLMP. Our proposed moderated mediation model reflects the complex relationships between constructs, which are linked to the following questions:

RQ1: Does GLKE affect GLP via the mediating role of GLMP?
RQ2: Does GIC moderate the indirect effects of GLKE on GLP via GLMP?

These research questions are linked to two research objectives: 1) to test the mediating effect of GLMP on the relationship between GLKE and GLP and 2) to test the moderating role of GIC on this mediating effect. To answer the above questions and achieve these objectives, the researchers surveyed 142 managers in Vietnamese logistics businesses and conducted an analysis using partial least squares structural equation modeling (PLS-SEM) with the SmartPLS v4.0.7.6 software. The research results contribute significantly to the current literature as follows: First, to our knowledge, this is the first study to explain the positive influence of GLKE on GLMP, implying that the implementation and application of green knowledge can support the practice of green management. Second, the researchers investigated the positive effect of GLMP on GLP and found that green logistics management can help Vietnamese logistics firms achieve environmental goals in logistics activities and improve competitiveness. Third, a critical contribution is the finding that GLKE indirectly influences GLP through the mediating role of GLMP. The exploitation and application of green knowledge positively and indirectly influence the achievement of environmental goals through active participation in environmental management activities. Finally, this study is novel in its exploration of the moderating role of GIC in the relationship between GLKE and GLMP, contributing to the scalability of the absorptive capacity theory, the resource-based view (RBV), and the resource orchestration theory. Logistics enterprises invest more in GIC, and GLKE contributes more to GLMP. Indeed, the application/exploitation of environmental knowledge (within and beyond the organization) can promote the use of sustainable vehicles and energy and aid in the evaluation of green activities in logistics firms [16,21]. This promotion is enhanced when logistics companies invest in employee training, the development of knowledge management systems, and the formation of collaborative relationships that shape the environment [[23], [24], [25]]. The results encourage logistics business managers to apply green logistics more quickly and effectively toward improving competitiveness and meeting stakeholder requirements.

## 2 Theoretical background and hypothesis development

### 2.1 Absorptive capacity theory

Cohen and Levinthal [26] introduced the absorptive capacity theory, which concerns the process of knowledge absorption. Absorptive capacity refers to recognizing the value of assimilation and applying the knowledge thus acquired in commercial activities; it plays a role in promoting innovation practices and improving performance. Later, Zahra and George [27] significantly expanded the process of Cohen and Levinthal [26] toward a higher competitive advantage, arguing that knowledge acquisition and assimilation affect knowledge transformation and exploitation, and that knowledge transformation combined with knowledge exploitation affects strategic flexibility, innovation, and performance. Todorova and Durisin [28] adjusted the process of absorptive capacity. Accordingly, knowledge exploitation serves as the endpoint of the process, directly promoting performance, innovation, and flexibility in management [28]. This view was applied in a green logistics case study by Abareshi and Molla [16], who suggested that green knowledge exploitation refers to an organization's ability to refine, extend, and manipulate existing or new green knowledge by combining acquired knowledge and converting it into green management practices.

Moreover, Dzhengiz and Niesten [29] show that the exploitation of external green knowledge not only develops the green capacity of managers but also promotes green governance in the organization toward a higher environmental responsibility. Concerning logistics activities, numerous studies have utilized the absorptive capacity theory to explain the capacity to convert/exploit the green knowledge of logistics enterprises into the process of environmentally oriented activities (e.g. Refs. [16,30]). To remain competitive, logistics companies must exploit and apply knowledge (including external knowledge, new knowledge, new ideas, and green knowledge) that promotes green management practices in purchasing and customer collaboration [31,32]. Moreover, based on the absorptive capacity theory, the researchers argue that externally acquiring, transforming, and utilizing green knowledge can assist logistics firms in achieving environmental objectives, such as improving production processes for recycling and using recycled materials, providing additional environment-related specifications of goods, and changing management processes toward the environment [33]. Regarding the theoretical insight into logistics operations, the relationship between GLKE and GLMP remains unexplored. Therefore, this study uses the absorptive capacity theory to propose the relationship between GLKE and GLMP.

### 2.2 RBV

The RBV pertains to how a business’s internal resources contribute to its sustainable competitive advantage [34]. When businesses possess valuable, irreplaceable, and inimitable resources, they can achieve superior performance that is not easily achieved by their competitors. Hart [35] proposed the natural resource-based view (NRBV), an extension of RBV, which claims that three key strategic capabilities are sources of competitive advantage: pollution prevention, product stewardship, and sustainable development (i.e., clean technology and the base of the pyramid) [36]. In other words, businesses can create a sustainable competitive advantage when they possess the resources to meet their environmental requirements. Several studies have relied on the NRBV to explore the role of green resources (e.g., GIC, human resource management, and innovation practices) in fostering environmental performance (e.g. Refs. [1,3,37]). Based on the RBV, several studies suggest that green logistics practices in logistics companies are valuable and inimitable resources and that this is the primary driver of environmental performance and green competitiveness (e.g. Refs. [8,21]). Depending on the logistics firm’s capacity, it can engage in high or low levels of green logistics management, such as reverse logistics; building green reward programs; providing green training for employees; monitoring and evaluating environmental policy practices; and employing green transportation, product packaging, and distribution [8,21]. The greater this level, the less difficult it is for businesses to achieve environmental performance and green competitiveness. Based on the NRBV, the researchers consider GLMP a green resource in an organization and explore the link between GLMP and GLP.

### 2.3 Resource orchestration theory

This study utilizes the resource orchestration theory [38,39] to employ GIC as a moderator in the relationship between GLKE and GLMP. The resource orchestration theory, an extended form of the RBV, is more concerned with the role of managers in sustainable operations and enhancing competitive advantages [39]; it claims that managers should rely on their management knowledge to develop an effective combination of resources to help their firms sustain a superior advantage over their competitors [39,40] and promote sustainable practices [4]. In other words, managers must mobilize resources and integrate them into an appropriate structure. Effective interactions between resources in a proper structure can better support management activities and increase sustainable competitive advantages [39]. Based on the RBV and NRBV, the researchers claim that GLKE, GIC, and GLMP are green resources because they have unique characteristics in each organization and are valuable and difficult to imitate [34]. In line with previous research (e.g. Refs. [23,[41], [42], [43]]), the researchers contend that GIC is a green resource that can control and coordinate the interactions between other similar fundamental resources in organizations. This argument relies on the resource orchestration theory to explain the mechanism of resource combination in the green supply chain, which is closely related to green logistics [44,45]. The effective utilization of internal and external green information leads to the improved processing and dissemination of such information in logistics firms [8,16], and this impact is heightened when the company has a robust environmental knowledge management system [25]. Based on the resource orchestration theory, the researchers hypothesize that GLKE can positively influence GLMP in logistics firms under the control of GIC.

### 2.4 Green logistics

The field of logistics presents an opportunity for the transportation industry to adopt a more environmentally conscious image, following a belief from the late 1980s that transportation modes, infrastructure, and traffic cause environmental degradation [46]. The terms "green logistics" and "reverse logistics" were first coined in 1980 [46]. Green logistics involves the production and distribution of goods in a socially and environmentally responsible manner and is defined as a set of supply chain management strategies aimed at reducing the ecological footprint of goods distribution with a focus on material handling, waste management, packaging, and transportation [13,47].

The main goals of green logistics are to reduce the environmental impact of logistics activities; reduce energy consumption and waste; and improve brand value, operational efficiency, and cost savings by reducing energy use [12,13,47]. To achieve these goals, green logistics activities focus on reducing carbon emissions, using environmentally friendly containers and packaging, promoting green transportation, and minimizing the environmental impact on the supply chain [8,9,48].

The implementation of green logistics requires logistics companies to comply with environmental regulations and adjust their use of natural resources to produce and distribute environmentally friendly goods and services, thereby reducing environmental pollution [49]. Green logistics is a crucial component of the green supply chain and a roadmap for sustainable development as it helps businesses optimize their supply chain by identifying and selecting environmentally friendly material suppliers, offering eco-friendly solutions, and implementing green transport systems for delivery to consumers [8,9].

According to a 2022 Globe Newswire report [48], the global green logistics market is projected to reach USD 1,481.5 billion by 2028, expanding at a compound annual growth rate of 6.1% over the forecast period, based on the sales, revenues, and strategies of the 20 largest logistics companies in the world. Green logistics is a key development trend in the logistics industry and the most anticipated and innovative trend for 2021 [48]. By adopting green logistics, logistics companies can become more competitive in the global supply chain and better serve consumers.

### 2.5 Hypotheses development

#### 2.5.1 GLKE and GLMP

Green knowledge can be acquired from the outside and then transformed and applied to green management practices [50]; therefore, organizations must regularly update and reassess their environmental activities, such as green delivery processes [51]. Furthermore, updating green knowledge helps organizations build commercial contracts that are better connected to the environment [51]. Learning and acquiring information related to customers' green needs is a top priority for organizations to promote environmental management activities such as product recycling, waste reduction, green knowledge training for employees, and product innovation [52]. In another study, Dzhengiz and Niesten [29] suggested that when managers effectively exploit green knowledge from outside, they can conduct environmental management activities more confidently. In agreement with this theory, Zhou et al. [53] state that when senior managers effectively exploit green knowledge, they can implement environmental management activities more actively (e.g., promulgating policies toward environmental protection, closely monitoring the organization's environmental performance, and requiring more environmental reports).

Consistent with Abareshi and Molla [16], this study uses the absorptive capacity theory adapted by Todorova and Durisin [28] to suggest a link between GLKE and GLMP. The exploitation of green knowledge can positively contribute to environmental strategies in logistics firms, and a growing number of businesses are taking advantage of this knowledge and applying it to green activities. This is considered a driving force for promoting GLMP by increasing the use of sustainable logistics tools (e.g., green transport, energy, packaging, and distribution) [8]. Moreover, green logistics knowledge can contribute more to social responsibility when an organization actively participates in reverse logistics. Additionally, the effective exploitation of external green logistics knowledge is related to providing green training for employees, allowing them to evaluate and monitor environmental practices in the organization [9,21]. Finally, the researchers believe that applying green knowledge supports the processing and distribution of green information in logistics networks. Based on the above arguments, the researchers propose the following hypotheses:

H1 GLKE positively relates to GLMP.

#### 2.5.2 GLMP and GLP

Managers are increasingly interested in green management activities because they demonstrate their ability to encourage businesses to achieve environmental goals. Several studies have shown that green supply chain management practices positively support environmental performance [54,55]. Meanwhile, as a function, the role of GLMP in environmental performance—which is a major part of green supply chain management—has only been explored in a few studies [8,9]. Accordingly, the GLMP refers to green principles and strategies integrated into logistics activities to conserve resources, reduce negative impacts on the environment and society [8], and help businesses sustain outstanding performance [9].

Consistent with previous studies [1,3,37], the researchers rely on the NRBV to explore the association between GLMP and GLP, and argue that when businesses engage in reverse logistics (focusing on reducing the impact of logistics on the environment), they can reduce the costs associated with environmental compliance. Moreover, the development of green reward programs and training can improve environmental ethics, compliance with environmental standards, and the green image of businesses [9,21]. Additionally, the researchers posit that the use of green transportation, packaging, and distribution, as well as sustainable energy, can reduce CO^2^ emissions and resource consumption and improve the environmental situation of businesses. Based on these arguments, the following hypotheses are proposed:

H2 GLMP positively relates to GLP.

#### 2.5.3 Mediating role of GLMP

According to the green flow theory proposed by Errmann et al. [56], green mindfulness is a source of pro-environmental behavior. Green knowledge is also believed to promote pro-environmental behavior (e.g., green innovation and green management practices) [57], which can facilitate environmental and social performance [1,21]. Therefore, the researchers argue that green knowledge indirectly affects environmental performance through the mediating role of green management practices.

The implementation of green logistics knowledge can improve employees' environmental ethics and compliance with environmental standards through green training while also promoting activities toward more environmental benefits [8,16]. With the effective exploitation and application of green information, businesses can make more efforts to reduce CO^2^ emissions and resource consumption by increasing the use of sustainable energy and green transportation, packaging, and distribution, as well as designing green reward programs. Active participation in reverse logistics can improve the environmental situation of enterprises, enhance their green image, and prompt great accolades from stakeholders [58]. Based on the above discussion, the following hypotheses are proposed:

H3 GLMP mediates the relationship between GLKE and GLP.

#### 2.5.4 Moderating role of GIC

Intellectual capital refers to the wealth of knowledge and intangible assets that can be used to create new value by transforming them into new methods, processes, products, and services [40]. In every enterprise, intellectual capital is considered a core resource for promoting innovation [59], improving operational efficiency [60], and enhancing competitive advantage [61]. From an environmental perspective, GIC refers to intangible resources, knowledge, capabilities, and relationships geared toward environmental protection and green innovation practices [62,63]. Indeed, GIC plays a vital role in promoting pro-environmental behaviors in organizations, such as limiting the use of non-renewable materials, saving electricity and water [37], increasing environmental training for employees, hiring staff with environmental knowledge, assessing environmental management practices [3,64], and using clean technology to prevent pollution [25].

According to Chen [62], GIC consists of three aspects: The first is green human capital, which refers to employees' knowledge, skills, abilities, experience, intelligence, creativity, and commitment to protecting the environment. The second is green structural capital, which is related to knowledge management systems, reward systems, information technology systems, databases, operating processes, management mechanisms, patents toward environmental protection, and green innovation practices [62,63]. The third aspect is green relational capital, which shows the interactive relationships between a business and its suppliers, customers, and partners in environmental protection and green innovation [64], helping companies take advantage of green relationship networks to create new opportunities and improve competitiveness [21].

Previous studies have confirmed the moderating role of intellectual capital in organizational performance-related activities (e.g. Ref. [65]). Other studies have paid more attention to the dimensions of intellectual capital, such as human capital (e.g. Ref. [66]) and relational capital (e.g. Ref. [43]). Kianto et al. [41] proved that when more intellectual capital is invested, the relationship between knowledge management practices and organizational performance becomes stronger. Additionally, numerous studies have also explored the moderating role of green structural capital, and green relational capital [23,24]. In this study, the researchers rely on the resource orchestration theory to investigate the moderating role of GIC for two reasons. First, employees' green skills and knowledge can be recorded and shared when the enterprise has a better knowledge management system and can make a positive contribution to green process innovation practices [42]; in contrast, if green knowledge is not used properly, it does not receive attention from the management. Thus, it is not deployed under a knowledge management system (i.e., it lacks investment in GIC), which can reduce the speed of green process innovation [63,67]. Second, based on Martín-de Castro et al.’s [68] study, the researchers argue that knowledge mining could contribute more positively to product innovation if the company invests more in human capital. Furthermore, Kianto et al. [41] suggest that knowledge management practices (application of technological knowledge, using patents) can positively affect a firm’s performance; this is more effective when the organization invests more in human capital (recruiting university graduates, equipping workers, and hiring external professionals to conduct training and coaching) [41].

This study was conducted in the logistics industry. According to the resource orchestration theory, effective interaction between green resources in an appropriate structure can better support green management practices [39,69]. The researchers argue that GLKE could positively contribute to GLMP under GIC moderation and that the success in exploiting green logistics information (both internal and external) can contribute to the ability to process and distribute green information in the logistics network [8]. This can happen when businesses maintain stable green relationships with strategic partners in the network; moreover, they can also increase the use of green transportation, packaging, and distribution [8,9] to link logistics activities with environmental benefits. This is easier when businesses have more green investments (e.g., building an environmental management system, designing operating procedures that are environmentally oriented, and improving employees’ environmental knowledge and the development capacity of green products and services). Additionally, the combination of employees' environmental competence and green support from managers can control the contribution of green knowledge implementation and application to training and coaching. Employees can monitor and evaluate green activities in an organization [8,9]. These arguments also reinforce the moderating capacity of GIC for the relationship between GLKE and GLMP. Based on the above discussion, the following hypotheses are proposed:

H4a GIC positively moderates the effect of GLKE on GLMP.

H4b GIC positively moderates the indirect effect of GLKE on GLP via GLMP

**Fig. 1:**
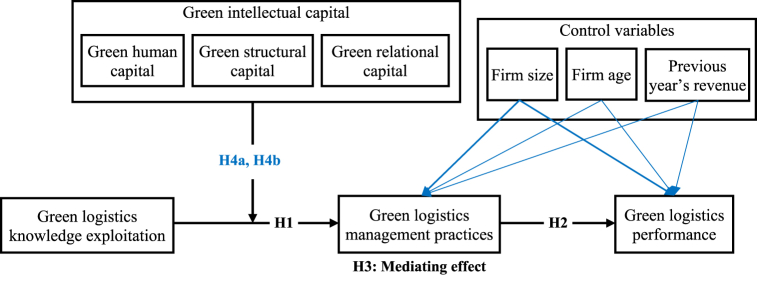
Proposed research model.

Fig. 1 illustrates the proposed model and its hypotheses.

## 3 Methodology

### 3.1 Research site

In Vietnam, logistics is a service industry that focuses on investments because of its important role in the economy. The Government of Vietnam issued Decision 221 QD-TTG to improve competitiveness and develop logistics services by 2025, expecting the contribution of logistics services to GDP to reach 5%–6%, which can be ranked 50th in the world or higher according to the Logistics Performance Index [70]. Furthermore, according to Agility’s (the world's leading logistics service provider) report on the logistics index in emerging markets, Vietnam is ranked 8th among the top 10 countries worldwide [20]. By the end of 2020, Vietnam had 35,744 logistics businesses, an increase of 5.5% compared to 2019. Most companies perform transportation functions (road, rail, sea, air, and inland waterway freight) such as forwarding, warehousing, packaging, and distribution. According to the VLMR [20], the primary revenue of Vietnam's logistics firms in 2020 included transportation with 11.07 billion USD (of which road freight accounted for more than 70%), warehousing with 11.71 billion, and forwarding with 512.33 million.

Vietnam has many advantages in becoming the logistics center of the Association of Southeast Asian Nations (ASEAN) because of its geographical location and long coastline (1.0 million square km of the sea). This area is located on the arterial traffic route connecting the Pacific-Indian Ocean, Europe-Asia, and Middle East Asia, and is considered the second busiest international transport route in the world. Vietnam has signed many trade agreements, forcing its manufacturing industry to restructure, open new markets, and attract international goods. Vietnam has also focused on the macroeconomic environment by investing more in transport infrastructure (570,448 km of roads, while the national highway network has reached 24,136 km; 297 railway stations) [20]. Moreover, there have been efforts to reform administrative procedures to facilitate trade in goods and increase investment attraction in the logistics market [20].

Among the operating logistics enterprises, 90% are Vietnamese, with only 30% of the market share, and 70% belong to foreign enterprises. Vietnam's logistics costs/GDP is 20%, which is relatively high compared to other countries in the region, such as Singapore, Thailand, Malaysia, and China, as well as the world average (10.8%) [20]. Most logistics enterprises in Vietnam operate traditionally; they are small (90% of logistics enterprises have capital of less than 430,000 USD) and face a shortage of human resources [20,71]. These limitations result in relatively low competitiveness. However, with the development trend of the logistics industry, green logistics is considered an effective solution to improve competitiveness in developing countries [6] by reducing CO^2^, noise, and waste, as well as increasing the use of green logistics vehicles and saving energy and, more importantly, costs [8,9,16]. In Vietnam, implementing green logistics is inevitable as the country transitions from traditional logistics toward improving its competitiveness [6,21]. Moreover, the implementation of green logistics contributes to environmental protection, promotes sustainable development, and meets the government’s efforts toward achieving the SDGs.

### 3.2 Data and sample

The sample consisted of Vietnamese logistics businesses. However, it is very difficult to obtain the contact details of all logistics businesses in Vietnam because this information is not available in any single report; therefore, the researchers sampled logistics businesses that are part of the Vietnam Logistics Business Association (VLA). This was the most feasible option, as the VLA contained complete information about its 646 members, including names, addresses, websites, emails, and phone numbers. Because enterprises in the VLA are from different regions (South, Central, and North Vietnam), they also represent the entire population. Furthermore, the VLA is also an official member of the International Federation of Freight Forwarders Associations (FIATA) and the ASEAN Federation of Forwarders Associations (AFFA); thus, the researchers assume that these businesses have professional logistics management systems.

The survey questionnaire was developed based on the scale items of the main constructs adopted from the literature, and back-translated using the procedure proposed by Brislin [72]. Following the recommendation of Agyabeng-Mensah et al. [8], it was tested in two phases: In phase one, the researchers sent the draft questionnaire to four experts working in a Vietnamese logistics research and development institute to check for content, clarity, and scalability, and made some revisions. In the second phase, the researchers sent the revised questionnaire via email to 20 logistics enterprises in the VLA to receive inputs regarding content, design, and usability, based on which the researchers made minor adjustments. The final questionnaire was found to be optimal because it was based on appropriate recommendations from both experts and managers.

The unit of analysis in this study is a logistics enterprise, and the survey respondents must be managers because they have sufficient knowledge and competence to complete the questionnaire on behalf of the organization, which is consistent with Agyabeng-Mensah et al. [8]. Using a convenient sampling approach, the researchers randomly generated a list of 500 businesses from the VLA. The researchers conducted an online survey at the beginning of April 2022 and sent questionnaires (with a reference letter for research purposes) via email to 500 businesses. All participants provided informed consent. Because the researchers did not request their names or the names of their companies, they were guaranteed complete confidentiality and anonymity. Reminders were sent every two weeks to improve the response rate. Two months after the survey (April–June 2022), the researchers received 171 responses from 171 businesses. Managers’ responses that were invalid or incomplete were excluded from the sample. Finally, responses from 142 managers were included in the analysis for a response rate of 28.4% (142/500). This is higher than the minimum response rate (20%) recommended for green supply chain studies [73]. In addition, there were 43, 55, and 44 logistics firms in the northern, central, and southern regions of Vietnam, respectively. This indicates that the sample firms are geographically representative. Table 1 presents the respondents’ profiles.

**Table 1:** Profile of respondents (n = 142).

Criteria	Frequency	Percent	Criteria	Frequency	Percent
*Gender*			*Firm age (years)*		
Male	49	34.5	Less than 5	29	20.4
Female	93	65.5	5–15	85	59.8
*Position*			16–25	20	14.1
General managers	65	45.7	Above 25	8	5.7
Planning and logistics managers	34	23.9	*Firm size (employees)*		
Maintenance managers	25	17.6	Less than 50	127	89.4
Factory managers	18	12.8	50–100	9	6.3
*Education*			101–150	4	2.8
High school	37	26.1	Above 150	2	1.5
Bachelor	87	61.1	*Previous year's revenue (US dollars)*		
Master	18	12.8	Less than 50,000	21	14.8
			50,001–200,000	100	70.4
			200,001–500,000	19	13.3
			Above 500,000	2	1.5

### 3.3 Non-response and common method bias

Based on the suggestion of Armstrong and Overton [74], this study tested for non-response bias by comparing respondents and non-respondents. The researchers performed this comparison based on multiple demographic characteristics. An independent *t*-test was used to test for differences between the two groups according to demographic characteristics. The results showed a p-value >0.05, indicating no statistically significant differences in the means between respondents and non-respondents. Thus, there was no evidence of non-response bias in our study.

Based on the recommendations of Podsakoff et al. [75], the researchers evaluated for common method bias (CMB) using Harman’s single-factor test. All items from every construct were loaded into a factor analysis to check whether a single general factor resulted in majority covariance among the measures. The generated principal component analysis output revealed that the first factor accounted for only 39.973% of the variance in the data (less than 50%), suggesting that CMB was not an issue in our study. Moreover, the researchers used the marker variable to check for CMB based on the recommendation of Lindell and Whitney [76], which was created using four items of the social desirability scale that had no theoretical relationship with any construct and appeared at the end of the questionnaire. CMB will occur when the correlation coefficient (r) between social desirability and any construct is higher than 0.3 [76]. The results from the PLS-Algorithm show that the maximum r values are 0.236 and 0.242 at the first order and second order, respectively. Thus, CMB is not a serious concern in our study. Next, the researchers partialled out the social desirability by adding endogenous latent variables to check for changes in R^2^. The results showed that the R^2^ (without social desirability) values of GLMP and GLP were 0.708 and 0.406, respectively, while the R^2^ (with social desirability) values of GLMP and GLP were 0.708 and 0.419, respectively. There was no significant difference in the R^2^ values of the endogenous constructs before and after the addition of social desirability. Thus, there was no risk of CMB in the present study.

### 3.4 Measures

Following previous empirical studies on green logistics [8,9,21], reflective scales were used to measure the main constructs. Valid measures were determined by reviewing the literature and adopting multi-item scales to measure GIC, including GHC, GSC, and GRC. The measurement scale was scored on a five-point Likert-type scale ranging from 1 (very low extent) to 5 (very high extent). The GIC was measured using the 11-item scale proposed by Chen [62] and Huang and Kung [77]. This measurement was also used in previous studies [25,64]. Next, GLKE was measured using five items built by Gluch et al. [78] and Lichtenthaler [79]. These items have also been used to measure the GKLE in research on green logistics in Australia [16]. A five-point Likert scale (from 1 = very low to 5 = very high) was used to measure GLKE. To measure GLP, nine items were developed based on previous studies [80,81]; Abareshi and Molla [16] used this measure. A five-point Likert scale (from 1 = very low to 5 = very high) was used to measure GLP. GLMP was measured using six items, as suggested in several previous studies [82]. This measurement method was also used by Agyabeng-Mensah et al. [8]. A five-point Likert scale (from 1 = very low to 5 = very high) was used to measure the GLMP.

Finally, firm size, age, and the previous year's revenue are included in the model as control variables. Larger businesses with longer operating times and higher previous year's revenues are more likely to implement green logistics management and promote environmental performance [9,21].

### 3.5 Data analysis

PLS-SEM with the SmartPLS v. 4.0.7.6 software was used to test the hypotheses. PLS-SEM is suitable for exploratory and experimental studies in the early stages of theory development, as well as for complicated models with moderating and mediating variables [83]. The small sample size of 142 was also acceptable for PLS-SEM analysis, as it is the optimal choice for such cases [84]. Indeed, the “10-times rule” is often used to determine the minimum sample size in the PLS-SEM approach [85]. Applying this principle to Fig. 1, the GLP (or GLMP) is directly affected by four variables; the minimum sample size should be greater than 40. However, Kock [86] has suggested that the minimum sample size proposed by Marcoulides and Saunders should be followed [87]. Thus, the maximum number of arrows for the GLP (or GLMP) is four, and the minimum sample size should be greater than 65. With 142 responses, the sample size satisfies these conditions.

## 4 Results

### 4.1 Measurement model

First, model fit was tested based on the standardized root mean square residual (SRMR). SmartPLS provides an SRMR of 0.062, which is lower than 0.08; thus, the data are suitable for the proposed model, and the path model has a good fit [88]. Table 2 shows that the outer loadings of the constructs are all higher than 0.708, which is satisfactory (ranging between 0.729 and 0.889) [89]. Reliability was tested using composite reliability (CR), and the results showed that the CR values ranged between 0.877 and 0.936, higher than 0.70. Therefore, all the measurement scales were reliable. Table 2 also shows that all constructs had an average variance extracted (AVE) higher than 0.50 (ranging between 0.617 and 0.742), and convergent validity was acceptable.

**Table 2:** Measurement of constructs (first order).

Constructs and their measure	Items	Outer loadings	CR	AVE
Green logistics knowledge exploitation [16]			0.934	0.740
We consider environmental issues in strategic decision making	GLKE1	0.844		
We easily implement new knowledge and technology into green practice	GLKE2	0.860		
We apply new knowledge and technology in green practice	GLKE3	0.889		
We strive to change our activities toward environmental benefits	GLKE4	0.821		
We can successfully exploit internal and external information and knowledge into specific applications	GLKE5	0.884		
Green logistics management practices [8]			0.924	0.669
We participate in reverse logistics practices	GLMP1	0.801		
We build green reward programs	GLMP2	0.841		
We provide green training for employees, monitor and evaluate environmental policy practices	GLMP3	0.850		
We use green transportation, product packaging, and distribution	GLMP4	0.817		
We use green energy	GLMP5	0.865		
We apply a green information processing and distribution process	GLMP6	0.729		
Green human capital [25]			0.896	0.742
Our employees have a better level of contribution to environmental protection than our competitors	GHC1	0.842		
In our company, the ability of employees towards environmental protection is better than that of competitors	GHC2	0.863		
Managers fully support employees in achieving environmental protection goals	GHC3	0.879		
Green structural capital [25]			0.904	0.654
Our environmental protection management system is superior to competitors	GSC1	0.796		
We invest in more environmentally friendly facilities than our competitors	GSC2	0.848		
We have a better capacity to develop green products than our competitors	GSC3	0.829		
We design operating processes for smooth environmental protection	GSC4	0.789		
We have an environmental knowledge management system designed to facilitate the accumulation of environmental knowledge	GSC5	0.779		
Green relational capital [25]			0.877	0.705
Our relationship with suppliers in protecting the environment is always stable	GRC1	0.795		
The relationship between our customers and us in protecting the environment is always stable	GRC2	0.872		
We have a good cooperative relationship in environmental protection with strategic partners	GRC3	0.849		
Green logistics performance [16]			0.936	0.617
The company reduces the overall environmental footprint	GLP1	0.770		
The company reduces CO^2^ emissions	GLP2	0.769		
The environmental situation is improved	GLP3	0.801		
The company reduces the costs of environmental compliance	GLP4	0.816		
The company reduces energy consumption	GLP5	0.743		
The company that improves the green brand value	GLP6	0.786		
The company improves the environmental ethics of employees	GLP7	0.808		
The company complies with government regulations	GLP8	0.796		
The company received great environmental accolades	GLP9	0.780		

**Notes:** GLKE: green logistics knowledge exploitation; GLMP: green logistics management practices; GHC: green human capital; GSC: green structural capital; GRC: green relational capital; GLP: green logistics performance; AVE: average variance extracted; CR: composite reliability.

The researchers tested discriminant validity using the Fornell-Larcker criterion and the heterotrait-monotrait ratio (HTMT). Table 3 shows that the square root values of the AVE ranged between 0.786 and 0.861, which were higher than the inter-construct correlations (ranging between 0.096 and 0.738). Moreover, the HTMT indices did not exceed the threshold of 0.90 (ranging between 0.123 and 0.871). These results confirm that all constructs have satisfactory discriminant validity.

**Table 3:** Discriminant validity.

	1	2	3	4	5	6
1. GHC	**0.861**					
2. GLKE	−0.096	**0.860**				
	*0.123*					
3. GLMP	0.538	0.503	**0.818**			
	*0.621*	*0.550*				
4. GLP	0.350	0.297	0.637	**0.786**		
	*0.399*	*0.317*	*0.691*			
5. GRC	0.551	0.409	0.738	0.558	**0.839**	
	*0.684*	*0.474*	*0.871*	*0.652*		
6. GSC	0.475	0.320	0.672	0.588	0.532	**0.809**
	*0.561*	*0.350*	*0.754*	*0.650*	*0.639*	

**Notes:** First value = correlation between variables (off diagonal); second value (italic) = HTMT ratio; numbers in bold diagonal: square root of average variance extracted; GLKE: green logistics knowledge exploitation; GLMP: green logistics management practices; GHC: green human capital; GSC: green structural capital; GRC: green relational capital; GLP: green logistics performance.

Table 4 presents the results of the discriminant validity analysis. The results show that all HTMT ratios (ranging between 0.317 and 0.876) were lower than 0.9. Therefore, all constructs had satisfactory discriminant validity.

**Table 4:** Discriminant validity analysis.

	1	2	3	4
1. GIC	**0.689**			
2. GLKE	0.278	**0.860**		
	*0.374*			
3. GLMP	0.788	0.503	**0.818**	
	*0.876*	*0.550*		
4. GLP	0.621	0.297	0.637	**0.786**
	*0.679*	*0.317*	*0.691*	

**Notes:** First value = correlation between variables (off diagonal); second value (italic) = HTMT ratio; numbers in bold diagonal: square root of average variance extracted; GLKE: green logistics knowledge exploitation; GLMP: green logistics management practices; GLP: green logistics performance.

### 4.2 Structural model

Three hierarchical models were designed to test the hypotheses (see Table 5). First, the researchers tested for multicollinearity using the variance inflation factor (VIF). The maximum inner variance inflation value was 1.426 (Model 3), which was significantly lower than Model 3 without multicollinearity [89]. Next, the researchers predicted the relationship between endogenous and exogenous variables through R-square (R^2^) and effect size (f^2^); accordingly, R^2^ higher than the threshold of 0.10 is acceptable [89], while Cohen [90] suggests that f^2^ is small, medium, and large, with f^2^ values of 0.02, 0.15, and 0.35, respectively. The minimum R^2^ was 0.256 (Model 2) and the maximum was 0.730 (Model 3), whereas the minimum f^2^_(GLKE→GLMP)_ was 0.319, and the minimum f^2^_(GLMP→GLP)_ was 0.674. Finally, the minimum f^2^_(GLKExGIC→GLMP)_ was 0.077. These indicators suggest a fairly good relationship between the main variables [89].

**Table 5:** Hypothesis testing results.

	Dependent variables	Model 1	Model 2 (with GLP as the mediating variable)		Model 3 (with GLP as the mediating variable and GIC as the moderating variable)	
		GLMP	GLMP	GLP	GLMP	GLP
Direct effects						
H1	GLKE	0.504 (6.129)***	0.502 (5.957)***		0.377 (7.162)***	
	GIC				0.726 (17.692)***	
H2	GLMP			0.635 (11.191)***		0.635 (10.969)***
Mediating effects						
H3	GLKE→GLMP→GLP			0.319 (5.845)***		0.239 (6.897)***
Moderating effects						
H4a	GLKE × GIC→GLMP				0.128 (3.313)***	
Control variables						
	SIZE	0.010 (0.115)^ns^	0.007 (0.089)^ns^	−0.048 (0.781)^ns^	−0.013 (0.307)ns	−0.047 (0.779)^ns^
	YEAR	0.002 (0.033)^ns^	0.003 (0.049)^ns^	0.039 (0.739)^ns^	0.012 (0.244)ns	0.039 (0.706)^ns^
	REV	−0.030 (0.466)^ns^	−0.031 (0.481)^ns^	−0.061 (0.984)^ns^	−0.035 (0.782)ns	−0.060 (0.949)^ns^
R^2^		0.257	0.256	0.415	0.730	0.413
Maximum Inner VIF	1.062	1.062	1.426			

**Notes:** Numbers in parentheses: t-value; GLKE: green logistics knowledge exploitation; GLMP: green logistics management practices; GLP: green logistics performance; SIZE: firm size; YEAR: firm age; REV: previous year's revenue; *** Significant at the 1% level; ^ns^ Not significant.

### 4.3 Hypothesis testing results

Table 5 shows that the direct effects are all statistically significant; in fact, GLKE has a positive effect on GLMP (β = 0.504, t = 6.129 (model 1); β = 0.502, t = 5.957 (model 2); β = 0.377, t = 7.162 (model 3)), while GLMP also positively affects GLP (β = 0.635, t = 11.191 (model 2); β = 0.635, t = 10.969 (model 3)). The study also confirms the indirect influence of GLKE on GLP through the mediating role of GLMP (β_indirect effect_ = 0.319, t = 5.845 (model 2); β_indirect effect_ = 0.239, t = 6.897 (model 3)). Hypotheses H1, H2, and H3 are supported.

**Fig. 2:**
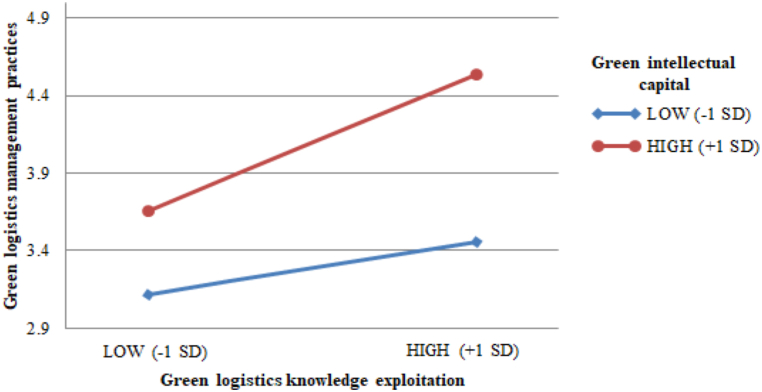
Interaction effect of GLKE with GIC on GLMP.

To test the moderating hypothesis, H4a, an interaction term was created (GLKE × GIC) from the mean centered on the independent variable (GLKE) and moderating variables (GIC) to avoid multicollinearity [91]. Table 5 shows that GIC positively moderates the relationship between GLKE and GLMP (β_GLKE×GIC→GLMP_ = 0.128, t = 3.313), indicating that when logistics enterprises invest more in GIC, the impact of GKLE on GLMP becomes stronger (see Fig. 2). Thus, hypothesis H4a was supported.

To examine H4b regarding the moderating effect of GIC on the indirect effect of GLKE on GLP via GLMP, the researchers employed PROCESS macro v3.4 model 7 [92] to compute the indirect effect of the independent variable (i.e., GLKE) on the dependent variable (i.e., GLP) at low (−1 SD), mean, and high (+1 SD) levels of the moderator (i.e., GIC) using 5,000 bootstrap samples. This approach is consistent with previous studies (e.g. Refs. [[93], [94], [95]]).

Table 6 demonstrates that all conditional indirect effects of GLKE on GLP via GLMP at low (−1 SD), mean, and high (+1 SD) levels of GIC were significant, given that the confidence intervals (ranging between 0.089 and 0.398) did not contain zero. Furthermore, the conditional indirect effect of GLKE on GLP via GLMP increased (from 0.160 to 0.283) when GIC increased (from −1 SD to +1 SD), indicating that GIC positively moderates the mediating effect of GLMP on the relationship between GLKE and GLP. Thus, H4b was supported.

**Table 6:** Conditional indirect effect of GLKE on GLP via GLMP.

GIC	Estimate	SE	LLCI	ULCI
−1 SD	0.160	0.036	0.089	0.231
Mean	0.239	0.044	0.158	0.333
+1 SD	0.283	0.054	0.183	0.398
Note: LLCI: lower limit confidence interval; ULCI: upper limit confidence interval; SE: standard error; SD: standard deviation				

## 5 Discussion

Based on the absorptive capacity theory, NRBV, and resource orchestration theory, this study designs a green logistics model for Vietnam, where logistics enterprises have relatively low levels of competitiveness [20]. Green logistics practices can be considered an effective solution to this problem [6,21]. Moreover, the application of green logistics is of interest to the Vietnamese government for supporting the SDGs. Accordingly, this study explores the direct, indirect, and moderate effects between constructs such as GLKE, GLMP, GLP, and GIC. As the first study to explore this phenomenon, the researchers confirm that GLKE has a positive effect on GLMP. Although green knowledge is an important factor in promoting competitiveness through green logistics practices in developing countries [6], only Abareshi and Molla [16] have explored its exploitation in the logistics industry. Our results fully support the absorptive capacity theory [28] in the context of the logistics industry in an emerging Asian market. Through training and coaching, logistics businesses can absorb, transform, and apply external green knowledge to their operations. Its main purpose is to increase the ability to control and evaluate environmental activities in an organization [8].

Moreover, the effective application of green knowledge supports the processing and distribution of green information in logistics networks and can improve organizations’ environmental consciousness. Consequently, they can increase their use of green transportation, packaging, distribution, and sustainable fuels, thus helping logistics enterprises reduce costs and improve revenue and competitiveness [21].

Hypothesis H2 was confirmed by the strong positive correlation between GLMP and GLP. Our results fully support the NRBV because, in line with Rehman et al. [96], the researchers considered green logistics management to be a green resource, and the results indicated that this green resource significantly promotes environmental performance [36]. Active participation in reverse logistics can reduce the costs associated with environmental compliance [8]. Furthermore, designing a green reward program and participating in green training can improve employees' environmental ethics while allowing the organization to be more compliant with environmental standards, which is useful for creating a green image of the company. In addition, the use of green transport and sustainable energy can reduce CO^2^ emissions and improve the environmental situation of enterprises [8,16,21].

Regarding the indirect effect, the study confirms that GLKE has an indirect impact on GLP through the mediating role of GLMP (H3). This discovery fits the context of the logistics industry in Vietnam, as logistics firms exploit green knowledge from outside and apply it to green management activities to achieve environmental performance or, in other words, to achieve environmental goals. The researchers believe that the implementation and application of green knowledge can improve employees' environmental ethics and compliance with environmental standards through green sharing, training, and coaching in the logistics industry. Moreover, the exploitation of green knowledge can raise logistics firms’ awareness of environmental activities; consequently, they can make greater efforts to reduce CO^2^ emissions and resource consumption by using green transportation, increasing green packaging and green distribution, and using fuel and green energy. Furthermore, even building/participating in green reward programs can enhance green logistics brands, prompting great accolades from stakeholders. This pertains to the effective application of green logistics knowledge in businesses.

The study confirms that GIC positively moderates the relationship between GLKE and GLMP as well as the indirect relationship between GLKE and GLP via GLMP (Hypotheses H4a and H4b are supported), which is also consistent with the resource orchestration theory. Thus, appropriate interactions among green resources in a suitable structure can promote green management activities [39]. As logistics enterprises invest more in GIC, the effect of GLKE on GLMP increases. The researchers argue that the acquisition, transformation, and application of green knowledge positively supports green training and coaching processes in logistics enterprises. Consequently, employees could evaluate and monitor green activities. This level of support is more robust when employees have higher environmental competence and receive better green support from managers. This is especially important in Vietnam, where human resources for the logistics industry are lacking [20]. Additionally, the successful exploitation of green logistics information (both internal and external) can contribute to the ability to process and distribute information within a logistics network. This contribution level increases when the organization maintains a stable green relationship with suppliers, customers, and strategic partners. The Vietnamese government is striving to build a green economy to achieve the SDGs, and green logistics practices can contribute to this goal. The government has also focused on the construction of infrastructure associated with the environment to support green logistics. Consequently, delivering and receiving goods becomes more convenient, and logistics businesses can maintain a stable information network (including green relationships) with stakeholders. Additionally, through cooperation with the FIATA, the AFFA can support green training in logistics enterprises in Vietnam, as they can learn and absorb green logistics knowledge from the outside. Subsequently, they can exploit or apply it to business activities associated with the environment to support green logistics management (such as processing and distributing green information in the logistics network) and improve competitiveness [6,21]. Support is stronger when logistics enterprises have more stable and greener relationships with stakeholders in the logistics network. Further, businesses can link logistics activities with environmental benefits by increasing green transportation, packaging, and distribution [8,9] through building an environmental management system, and improving employees’ environmental knowledge. Having a green knowledge management system can also make exploiting external green information more supportive of providing training and coaching employees, which can encourage businesses to achieve their environmental goals [42].

Further findings showed that GIC had a strong positive effect on the GLMP (Table 6). As GIC is considered a green resource, this result fully supports the NRBV. Therefore, they can help organizations achieve their environmental goals [62,63]. This result is also supported if GLMP is considered pro-environmental behavior; in fact, some previous studies (e.g. Refs. [3,25,37]) suggest that GIC promotes pro-environmental behavior, such as green innovation practices, business sustainability practices, or green human resource management practices.

Finally, in contrast to previous studies [8,9], the control variables (firm year, firm size, and previous year's revenue) did not affect GLMP or GLP. The researchers believe that Vietnamese logistics enterprises are transitioning from traditional logistics to green logistics. Therefore, enterprise characteristics do not yet reflect their relationships with green logistics practices. For example, businesses may not yet deduct the previous year's revenue from investing in green logistics because they may review and evaluate the effectiveness of green logistics before investing.

## 6 Conclusion

### 6.1 Theoretical contributions

Our research contributes to the existing literature in three ways. First, the researchers provide empirical evidence proving the plausibility of the NRBV theory and confirm a positive relationship between GLMP and GLP. In logistics enterprises, GLMP are considered necessary green resources for achieving environmental performance. This result is supported by previous studies on green logistics in Ghana [8,9]. Our findings show a positive association between GIC and GLMP (Table 6), which is also consistent with the NRBV theory. Previous studies have also employed the NRBV to confirm the relationship between GIC and pro-environmental behaviors, such as green innovation practices [25], business sustainability practices [37], and green human resource management practices [3]. This suggests that the NRBV theory is not geographically restricted to developing countries such as Vietnam. Researchers can extend the applicability of this theory to other research settings.

Second, the study broadens our understanding of the absorptive capacity theory, as it examines the direct relationship between the exploitation of green knowledge and competitive advantage [16] while ignoring the mediating mechanism of pro-environmental behavior. This research overcomes the limitations of Abareshi and Molla’s [16] study by demonstrating that GLMP mediates the relationship between GLKE and GLP. Implementing green knowledge can improve employees’ environmental ethics in the logistics industry through green training and coaching. Moreover, exploiting green knowledge can enhance organizations' awareness of environmental activities; consequently, they can make greater efforts to reduce CO^2^ emissions and resource consumption by increasing the use of green transport, distribution, and energy.

Third, by referring to the moderating role of GIC, this study adds to our understanding of the resource orchestration theory. Previous studies based on the theory have only considered GIC as an explanatory role for green behaviors, such as environmental performance measurement practices [5] or management accounting usage [4]. In the context of Vietnamese enterprise logistics, this study demonstrates the moderating role of GIC in the relationship between GLKE and GLMP; this is a pioneer study in diversifying the role of GIC with environmentally friendly activities based on the resource orchestration theory. This finding also opens a new research direction on the moderating role of GIC, which needs to be explored in future studies.

### 6.2 Practical implications

Through this study, managers of Vietnamese logistics firms will gain a better understanding of green logistics, enabling them to accelerate their implementation. The researchers put forward the following practical implications based on our results. First, our research shows that GLKE has a positive effect on GLMP, indicating that logistics enterprises should apply green knowledge more in strategic decisions, as it can help them align their activities toward environmental benefits. Moreover, the exploitation of green knowledge occurs not only within the organization but also outside, that is, among suppliers, customers, or strategic partners.

Second, this study confirmed the positive relationship between GLMP and GLP. The researchers argue that this effect has practical implications for improving competitiveness because it is considered an indicator of competitive advantage [16,21]. To achieve this, enterprises should actively participate in reverse logistics practices, businesses should provide green training for employees to monitor and evaluate environmental policies in the organization, and managers should issue regulations on green transportation, packaging, distribution, and energy use. Green reward policies are necessary when businesses require employees to contribute to green logistics. Additionally, applying green information processing and distribution systems can better support green logistics management.

Third, the researchers recommend that logistics businesses focus more on exploiting green knowledge because the results show that GLKE indirectly influences GLP through its mediating mechanism. Implementing green knowledge can help businesses improve their environmental situation, reduce energy consumption, and create green images through effective logistics management mechanisms. Businesses should also focus on green training and coaching for employees so that they can easily apply their knowledge to their operations; this improves employees’ environmental ethics and increases compliance with environmental standards.

Finally, businesses should invest more in GIC because it positively moderates the relationship between GLKE and GLMP; this also applies to Vietnam's logistics industry. If more attention is given to the GIC, the GLKE has a more positive impact on the GLMP. Through GLMP, logistics businesses can achieve their environmental goals and improve their competitiveness [21].

### 6.3 Limitations and future research directions

Despite its essential contributions to theory and practice, this study has several limitations. First, the sample size was small (only 142 managers), whereas the VLA included 646 logistics enterprises, which partly affected our findings. Second, this study was conducted in Vietnam and most respondents were from small businesses. Therefore, these results may not be generalizable. Future studies should be conducted in other countries to obtain additional insights that could add to our findings. Third, subsequent research should expand the number of survey participants to include logistics staff because they are directly involved in logistics activities and can therefore provide more reasonable opinions and assessments of green logistics. This could also contribute to an increase in the sample size of the study. Fourth, as the analysis results confirm the moderating role of GIC, future research should consider moderating roles based on GIC dimensions (green human, structural, and relational capital) for a more comprehensive assessment of the impact of GIC on environmental activities [4,5,97]. Finally, further research can augment our model by adding constructs such as competitive advantage or green competition to test whether green logistics practices promote competitiveness in developing economies.

## Author contribution statement

Vo Van Hien: Conceived and designed the experiments; Performed the experiments; Analyzed and interpreted the data; Contributed reagents, materials, analysis tools or data; Wrote the paper.

Nguyen Phong Nguyen: Conceived and designed the experiments; Performed the experiments; Analyzed and interpreted the data; Contributed reagents, materials, analysis tools or data; Wrote the paper.

## Funding statement

This work was supported by 10.13039/100019455University of Economics Ho Chi Minh City (UEH).

## Data availability statement

Data will be made available on request.

## Additional information

Supplementary content related to this article has been published online at [URL].

## Declaration of competing interest

The authors declare that they have no known competing financial interests or personal relationships that could have appeared to influence the work reported in this paper.

## References

[1] Rehman S.U., Kraus S., Shah S.A., Khanin D., Mahto R.V. (2021). Analyzing the relationship between green innovation and environmental performance in large manufacturing firms. Technol. Forecast. Soc. Change.

[2] Nguyen N.P., Adomako S. (2022). International orientation and environmental performance in Vietnamese exporting small‐and medium‐sized enterprises. Bus. Strat. Environ..

[3] Haldorai K., Kim W.G., Garcia R.F. (2022). Top management green commitment and green intellectual capital as enablers of hotel environmental performance: the mediating role of green human resource management. Tourism Manag..

[4] Asiaei K., Bontis N., Alizadeh R., Yaghoubi M. (2022). Green intellectual capital and environmental management accounting: natural resource orchestration in favor of environmental performance. Bus. Strat. Environ..

[5] Asiaei K., Jusoh R., Barani O., Asiaei A. (2022). How does green intellectual capital boost performance? The mediating role of environmental performance measurement systems. Bus. Strat. Environ..

[6] Afum E., Agyabeng-Mensah Y., Baah C., Asamoah G., Kusi L.Y. (2022). Eco-market orientation in the logistics industry: a conveyor belt for achieving organizational outcomes via green logistics practices. Int. J. Logist. Manag..

[7] Mohsin A.K.M., Tushar H., Hossain S.F.A., Chisty K.K.S., Iqbal M.M., Kamruzzaman M., Rahman S. (2022). Green logistics and environment, economic growth in the context of the Belt and Road Initiative. Heliyon.

[8] Agyabeng-Mensah Y., Afum E., Ahenkorah E. (2020). Exploring financial performance and green logistics management practices: examining the mediating influences of market, environmental and social performances. J. Clean. Prod..

[9] Agyabeng-Mensah Y., Afum E., Acquah I.S.K., Dacosta E., Baah C., Ahenkorah E. (2020). The role of green logistics management practices, supply chain traceability and logistics ecocentricity in sustainability performance. Int. J. Logist. Manag..

[10] Moh’d Anwer A.S. (2022). An investigation of transportation logistics strategy on manufacturing supply chain responsiveness in developing countries: the mediating role of delivery reliability and delivery speed. Heliyon.

[11] Li X., Liu D., Zhang Z., Cheng T., Liu L., Yuan J. (2022). The impact of internal and external green supply chain management activities on performance improvement: evidence from the automobile industry. Heliyon.

[12] Rodrigue J.P., Slack B., Comtois C. (2013).

[13] Sbihi A., Eglese R.W. (2010). Combinatorial optimization and green logistics. Ann. Oper. Res..

[14] Lee S.Y., Klassen R.D. (2008). Drivers and enablers that foster environmental management capabilities in small‐and medium‐sized suppliers in supply chains. Prod. Oper. Manag..

[15] Lai K.H., Wong C.W. (2012). Green logistics management and performance: some empirical evidence from Chinese manufacturing exporters. Omega.

[16] Abareshi A., Molla A. (2013). Greening logistics and its impact on environmental performance: an absorptive capacity perspective. Int. J. Logist. Res. Appl..

[17] Bag S., Gupta S. (2019). Examining the effect of green human capital availability in adoption of reverse logistics and remanufacturing operations performance. Int. J. Manpow..

[18] Karaman A.S., Kilic M., Uyar A. (2020). Green logistics performance and sustainability reporting practices of the logistics sector: the moderating effect of corporate governance. J. Clean. Prod..

[19] Seroka-Stolka O. (2014). The development of green logistics for implementation sustainable development strategy in companies. Procedia-Social Behav. Sci..

[20] (2021). Vietnam Logistics Market Report – VLMR.

[21] Agyabeng-Mensah Y., Tang L. (2021). The relationship among green human capital, green logistics practices, green competitiveness, social performance and financial performance. J. Manuf. Technol. Manag..

[22] Guo Y., Wang L., Yang Q. (2020). Do corporate environmental ethics influence firms’ green practice? The mediating role of green innovation and the moderating role of personal ties. J. Clean. Prod..

[23] Yu Y., Huo B. (2019). The impact of environmental orientation on supplier green management and financial performance: the moderating role of relational capital. J. Clean. Prod..

[24] Amores-Salvadó J., Cruz-González J., Delgado-Verde M., González-Masip J. (2021). Green technological distance and environmental strategies: the moderating role of green structural capital. J. Intellect. Cap..

[25] Wang C.H., Juo W.J. (2021). An environmental policy of green intellectual capital: green innovation strategy for performance sustainability. Bus. Strat. Environ..

[26] Cohen W.M., Levinthal D.A. (1990). Absorptive capacity: a new perspective on learning and innovation. Adm. Sci. Q..

[27] Zahra S.A., George G. (2002). Absorptive capacity: a review, reconceptualization, and extension. Acad. Manag. Rev..

[28] Todorova G., Durisin B. (2007). Absorptive capacity: valuing a reconceptualization. Acad. Manag. Rev..

[29] Dzhengiz T., Niesten E. (2020). Competences for environmental sustainability: a systematic review on the impact of absorptive capacity and capabilities. J. Bus. Ethics.

[30] Lee E.S., Song D.W. (2015). The effect of shipping knowledge and absorptive capacity on organizational innovation and logistics value. Int. J. Logist. Manag..

[31] Teixeira A.A., Jabbour C.J.C., de Sousa Jabbour A.B.L., Latan H., De Oliveira J.H.C. (2016). Green training and green supply chain management: evidence from Brazilian firms. J. Clean. Prod..

[32] Hong J., Zheng R., Deng H., Zhou Y. (2019). Green supply chain collaborative innovation, absorptive capacity and innovation performance: evidence from China. J. Clean. Prod..

[33] Zhang W., Zhang X., Zhou Q. (2021). How does knowledge seeking and knowledge generation promote green supply chain management? An empirical study from China. Int. J. Logist. Res. Appl..

[34] Barney J. (1991). Firm resources and sustained competitive advantage. J. Manag..

[35] Hart S.L. (1995). A natural-resource-based view of the firm. Acad. Manag. Rev..

[36] Hart S.L., Dowell G. (2011). Invited editorial: a natural-resource-based view of the firm: fifteen years after. J. Manag..

[37] Yusoff Y.M., Omar M.K., Zaman M.D.K., Samad S. (2019). Do all elements of green intellectual capital contribute toward business sustainability? Evidence from the Malaysian context using the Partial Least Squares method. J. Clean. Prod..

[38] Sirmon D.G., Hitt M.A., Ireland R.D. (2007). Managing firm resources in dynamic environments to create value: looking inside the black box. Acad. Manag. Rev..

[39] Sirmon D.G., Hitt M.A., Ireland R.D., Gilbert B.A. (2011). Resource orchestration to create competitive advantage: breadth, depth, and life cycle effects. J. Manag..

[40] Rehman S.U., Bresciani S., Ashfaq K., Alam G.M. (2022). Intellectual capital, knowledge management and competitive advantage: a resource orchestration perspective. J. Knowl. Manag..

[41] Kianto A., Ritala P., Spender J.C., Vanhala M. (2014). The interaction of intellectual capital assets and knowledge management practices in organizational value creation. J. Intellect. Cap..

[42] Jirakraisiri J., Badir Y.F., Frank B. (2021). Translating green strategic intent into green process innovation performance: the role of green intellectual capital. J. Intellect. Cap..

[43] Ritala P., Baiyere A., Hughes M., Kraus S. (2021). Digital strategy implementation: the role of individual entrepreneurial orientation and relational capital. Technol. Forecast. Soc. Change.

[44] Wong C.W., Wong C.Y., Boon‐itt S. (2018). How does sustainable development of supply chains make firms lean, green and profitable? A resource orchestration perspective. Bus. Strat. Environ..

[45] Yoo J.J.E., Cho M. (2021). Supply chain flexibility fit and green practices: evidence from the event industry. Int. J. Contemp. Hospit. Manag..

[46] Byrne P., Deeb A. (1993). Logistics must meet “green” challenge. Transport. Distrib.

[47] Seroka-Stolka O., Ociepa-Kubicka A. (2019). Green logistics and circular economy. Transport. Res. Procedia.

[48] (2022). Globe Newswire’s Report.

[49] Teixeira C.R.B., Assumpção A.L., Correa A.L., Savi A.F., Prates G.A. (2018). The contribution of green logistics and sustainable purchasing for green supply chain management. Indepen. J. Manag. Prod..

[50] Aboelmaged M., Hashem G. (2019). Absorptive capacity and green innovation adoption in SMEs: the mediating effects of sustainable organisational capabilities. J. Clean. Prod..

[51] Albort-Morant G., Leal-Rodríguez A.L., De Marchi V. (2018). Absorptive capacity and relationship learning mechanisms as complementary drivers of green innovation performance. J. Knowl. Manag..

[52] Awan U., Arnold M.G., Gölgeci I. (2021). Enhancing green product and process innovation: towards an integrative framework of knowledge acquisition and environmental investment. Bus. Strat. Environ..

[53] Zhou M., Govindan K., Xie X., Yan L. (2021). How to drive green innovation in China's China's mining enterprises? Under the perspective of environmental legitimacy and green absorptive capacity. Resour. Pol..

[54] Famiyeh S., Adaku E., Amoako-Gyampah K., Asante-Darko D., Amoatey C.T. (2018). Environmental management practices, operational competitiveness and environmental performance: empirical evidence from a developing country. J. Manuf. Technol. Manag..

[55] Wang Z., Wang Q., Zhang S., Zhao X. (2018). Effects of customer and cost drivers on green supply chain management practices and environmental performance. J. Clean. Prod..

[56] Errmann A., Kim J., Lee D.C., Seo Y., Lee J., Kim S.S. (2021). Mindfulness and pro-environmental hotel preference. Ann. Tourism Res..

[57] Martínez-Martínez A., Cegarra-Navarro J.G., Garcia-Perez A., Vicentini F. (2020). Extending structural capital through pro-environmental behaviour intention capital: an outlook on Spanish hotel industry. J. Intellect. Cap..

[58] Baah C., Jin Z., Tang L. (2020). Organizational and regulatory stakeholder pressures friends or foes to green logistics practices and financial performance: investigating corporate reputation as a missing link. J. Clean. Prod..

[59] Kianto A., Sáenz J., Aramburu N. (2017). Knowledge-based human resource management practices, intellectual capital and innovation. J. Bus. Res..

[60] Smriti N., Das N. (2018). The impact of intellectual capital on firm performance: a study of Indian firms listed in COSPI. J. Intellect. Cap..

[61] Yaseen S.G., Dajani D., Hasan Y. (2016). The impact of intellectual capital on the competitive advantage: applied study in Jordanian telecommunication companies. Comput. Hum. Behav..

[62] Chen Y.S. (2008). The positive effect of green intellectual capital on competitive advantages of firms. J. Bus. Ethics.

[63] Chang C.H., Chen Y.S. (2012). The determinants of green intellectual capital. Manag. Decis..

[64] Yong J.Y., Yusliza M.Y., Ramayah T., Fawehinmi O. (2019). Nexus between green intellectual capital and green human resource management. J. Clean. Prod..

[65] Mention A.L., Bontis N. (2013). Intellectual capital and performance within the banking sector of Luxembourg and Belgium. J. Intellect. Cap..

[66] Bornay‐Barrachina M., De la Rosa‐Navarro D., López‐Cabrales A., Valle‐Cabrera R. (2012). Employment relationships and firm innovation: the double role of human capital. Br. J. Manag..

[67] Zhou K.Z., Wu F. (2010). Technological capability, strategic flexibility, and product innovation. Strat. Manag. J..

[68] Martín-de Castro G., Delgado-Verde M., Navas-López J.E., Cruz-González J. (2013). The moderating role of innovation culture in the relationship between knowledge assets and product innovation. Technol. Forecast. Soc. Change.

[69] Asiaei K., Rezaee Z., Bontis N., Barani O., Sapiei N.S. (2021). Knowledge assets, capabilities and performance measurement systems: a resource orchestration theory approach. J. Knowl. Manag..

[70] Prime Minister (2021). https://luatvietnam.vn/thuong-mai/quyet-dinh-221-qd-ttg-sua-quyet-dinh-200-qd-ttg-phat-trien-dich-vu-logistics-198803-d1.html.

[71] Vietnam Briefing (2020). https://www.vietnam-briefing.com/news/vietnams-logistics-industry-how-vietnams-expanding-economy-boosting-growth.html/.

[72] Brislin R.W. (1970). Back-translation for cross-cultural research. J. Cross Cult. Psychol..

[73] Cankaya S.Y., Sezen B. (2018). Effects of green supply chain management practices on sustainability performance. J. Manuf. Technol. Manag..

[74] Armstrong J.S., Overton T.S. (1977). Estimating nonresponse bias in mail surveys. J. Market. Res..

[75] Podsakoff P.M., MacKenzie S.B., Lee J.Y., Podsakoff N.P. (2003). Common method biases in behavioral research: a critical review of the literature and recommended remedies. J. Appl. Psychol..

[76] Lindell M.K., Whitney D.J. (2001). Accounting for common method variance in cross-sectional research designs. J. Appl. Psychol..

[77] Huang C.L., Kung F.H. (2011). Environmental consciousness and intellectual capital management: evidence from Taiwan's manufacturing industry. Manag. Decis..

[78] Gluch P., Gustafsson M., Thuvander L. (2009). An absorptive capacity model for green innovation and performance in the construction industry. Construct. Manag. Econ..

[79] Lichtenthaler U. (2009). Absorptive capacity, environmental turbulence, and the complementarity of organizational learning processes. Acad. Manag. J..

[80] Zhu Q., Sarkis J. (2007). The moderating effects of institutional pressures on emergent green supply chain practices and performance. Int. J. Prod. Res..

[81] Darnall N. (2009). Regulatory stringency, green production offsets, and organizations' financial performance. Publ. Adm. Rev..

[82] Longoni A., Luzzini D., Guerci M. (2018). Deploying environmental management across functions: the relationship between green human resource management and green supply chain management. J. Bus. Ethics.

[83] Hair J.F., Hult G.T.M., Ringle C.M., Sarstedt M., Danks N.P., Ray S. (2021). Partial Least Squares Structural Equation Modeling (PLS-SEM) Using R, Springer Cham.

[84] Sarstedt M., Hair J.F., Ringle C.M. (2022). PLS-SEM: indeed a silver bullet"–retrospective observations and recent advances. J. Market. Theor. Pract..

[85] Lin H.M., Lee M.H., Liang J.C., Chang H.Y., Huang P., Tsai C.C. (2020). A review of using partial least square structural equation modeling in e‐learning research. Br. J. Educ. Technol..

[86] Kock N., Hadaya P. (2018). Minimum sample size estimation in PLS‐SEM: the inverse square root and gamma‐exponential methods. Inf. Syst. J..

[87] Marcoulides G.A., Saunders C. (2006). Editor's comments: PLS: a silver bullet?. MIS Q..

[88] Henseler J. (2017). Bridging design and behavioral research with variance-based structural equation modeling. J. Advert..

[89] Hair J.F., Risher J.J., Sarstedt M., Ringle C.M. (2019). When to use and how to report the results of PLS-SEM. Eur. Bus. Rev..

[90] Cohen J. (1988).

[91] Aiken L.S., West S.G., Reno R.R. (1991).

[92] Hayes A.F. (2018). Partial, conditional, and moderated moderated mediation: quantification, inference, and interpretation. Commun. Monogr..

[93] Abualigah A., Koburtay T., Bourini I., Badar K., Gerged A.M. (2023).

[94] Jain N.K., Panda A., Choudhary P. (2020). Institutional pressures and circular economy performance: the role of environmental management system and organizational flexibility in oil and gas sector. Bus. Strat. Environ..

[95] Srinivasan R., Swink M. (2018). An investigation of visibility and flexibility as complements to supply chain analytics: an organizational information processing theory perspective. Prod. Oper. Manag..

[96] Rehman S.U., Elrehail H., Alsaad A., Bhatti A. (2022). Intellectual capital and innovative performance: a mediation-moderation perspective. J. Intellect. Cap..

[97] Asiaei K., O'Connor N.G., Barani O., Joshi M. (2023). Green intellectual capital and ambidextrous green innovation: the impact on environmental performance. Bus. Strat. Environ..

