# Supplementary material for: Greening the Vietnamese supply chain: The influence of green logistics knowledge and intellectual capital
Source: Heliyon. 2023 Apr 29;9(5):e15953. doi: 10.1016/j.heliyon.2023.e15953 (PMC10165402; doi:10.1016/j.heliyon.2023.e15953)
Supplement: Multimedia component 1 [file mmc1.docx]

**Appendix**

**Section A. Background information**

*Individual information*

Gender

🗖 Male 🗖 Female

Education

🗖 High school 🗖 Bachelor

🗖 Master

Position

🗖 General managers 🗖 Planning & logistics managers

🗖 Maintenance managers 🗖 Factory managers

*Organizational information*

Age (years):…………..……………………………………………………………

Number of employees in your firm:……………………….……………………...

Previous year's revenue: ………………………………………………………….

**Section B. Questionnaire**

The following questions were answered based on perceptions with 5 levels: from 1 - strongly disagree to 5 – strongly.

| For each statement about **"green logistics knowledge exploitation"**, please indicate the extent of your agreement by selecting the box that reflects your current view of your expectation as a whole | | | | | |
| --- | --- | --- | --- | --- | --- |
|  | We consider environmental issues in strategic decision making | | | | |
|  | ⭘ | ⭘ | ⭘ | ⭘ | ⭘ |
|  | We easily implement new knowledge and technology into green practice | | | | |
|  | ⭘ | ⭘ | ⭘ | ⭘ | ⭘ |
|  | We apply new knowledge and technology in green practice | | | | |
|  | ⭘ | ⭘ | ⭘ | ⭘ | ⭘ |
|  | We strive to change our activities toward environmental benefits | | | | |
|  | ⭘ | ⭘ | ⭘ | ⭘ | ⭘ |
|  | We can successfully exploit internal and external information and knowledge into specific applications | | | | |
|  | ⭘ | ⭘ | ⭘ | ⭘ | ⭘ |
| For each statement about **"green logistics management practices"**, please indicate the extent of your agreement by selecting the box that reflects your current view of your expectation as a whole | | | | | |
|  | We participate in reverse logistics practices | | | | |
|  | ⭘ | ⭘ | ⭘ | ⭘ | ⭘ |
|  | We build green reward programs | | | |  |
|  | ⭘ | ⭘ | ⭘ | ⭘ | ⭘ |
|  | We provide green training for employees, monitor and evaluate environmental policy practices | | | | |
|  | ⭘ | ⭘ | ⭘ | ⭘ | ⭘ |
|  | We use green transportation, product packaging and distribution | | | | |
|  | ⭘ | ⭘ | ⭘ | ⭘ | ⭘ |
|  | We use green energy | | |  |  |
|  | ⭘ | ⭘ | ⭘ | ⭘ | ⭘ |
|  | We apply a green information processing and distribution process | | | | |
|  | ⭘ | ⭘ | ⭘ | ⭘ | ⭘ |
| For each statement about **"green human capital"**, please indicate the extent of your agreement by selecting the box that reflects your current view of your expectation as a whole | | | | | |
|  | Our employees have a better level of contribution to environmental protection than our competitors | | | | |
|  | ⭘ | ⭘ | ⭘ | ⭘ | ⭘ |
|  | In our company, the ability of employees towards environmental protection is better than that of competitors | | | | |
|  | ⭘ | ⭘ | ⭘ | ⭘ | ⭘ |
|  | Managers fully support employees in achieving environmental protection goals | | | | |
|  | ⭘ | ⭘ | ⭘ | ⭘ | ⭘ |
| For each statement about **"green structural capital"**, please indicate the extent of your agreement by selecting the box that reflects your current view of your expectation as a whole | | | | | |
|  | The environmental protection management system is superior to competitors | | | | |
|  | ⭘ | ⭘ | ⭘ | ⭘ | ⭘ |
|  | We invest in more environmentally friendly facilities than our competitors | | | | |
|  | ⭘ | ⭘ | ⭘ | ⭘ | ⭘ |
|  | We have a better capacity to develop green products than our competitors | | | | |
|  | ⭘ | ⭘ | ⭘ | ⭘ | ⭘ |
|  | We design operating processes toward smoothly environmental protection | | | | |
|  | ⭘ | ⭘ | ⭘ | ⭘ | ⭘ |
|  | We have an environmental knowledge management system designed to facilitate the accumulation of environmental knowledge | | | | |
|  | ⭘ | ⭘ | ⭘ | ⭘ | ⭘ |
| For each statement about **"green relational capital"**, please indicate the extent of your agreement by selecting the box that reflects your current view of your expectation as a whole | | | | | |
|  | Our relationship with suppliers in protecting the environment is always stable | | | | |
|  | ⭘ | ⭘ | ⭘ | ⭘ | ⭘ |
|  | The relationship between customers and us in protecting the environment is always stable | | | | |
|  | ⭘ | ⭘ | ⭘ | ⭘ | ⭘ |
|  | We have a good cooperative relationship in environmental protection with strategic partners | | | | |
|  | ⭘ | ⭘ | ⭘ | ⭘ | ⭘ |
| For each statement about **"green logistics performance"**, please indicate the extent of your agreement by selecting the box that reflects your current view of your expectation as a whole | | | | | |
|  | The company reduces the overall environmental footprint | | | | |
|  | ⭘ | ⭘ | ⭘ | ⭘ | ⭘ |
|  | The company reduces CO^2^ emissions | | | |  |
|  | ⭘ | ⭘ | ⭘ | ⭘ | ⭘ |
|  | The environmental situation is improved | | | |  |
|  | ⭘ | ⭘ | ⭘ | ⭘ | ⭘ |
|  | The company reduces the costs of environmental compliance | | | | |
|  | ⭘ | ⭘ | ⭘ | ⭘ | ⭘ |
|  | The company reduces energy consumption | | | | |
|  | ⭘ | ⭘ | ⭘ | ⭘ | ⭘ |
|  | The company that improves the green brand value | | | | |
|  | ⭘ | ⭘ | ⭘ | ⭘ | ⭘ |
|  | The company improves the environmental ethics of employees | | | | |
|  | ⭘ | ⭘ | ⭘ | ⭘ | ⭘ |
|  | The company complies with government regulations | | | | |
|  | ⭘ | ⭘ | ⭘ | ⭘ | ⭘ |
|  | The company received great environmental accolades | | | | |
|  | ⭘ | ⭘ | ⭘ | ⭘ | ⭘ |
| For each statement about **"social desirability"**, please indicate the extent of your agreement by selecting the box that reflects your current view of your expectation as a whole | | | | | |
|  | I’m always willing to admit it when I make a mistake | | | | |
|  | ⭘ | ⭘ | ⭘ | ⭘ | ⭘ |
|  | I never resent being asked to return a favor | | | | |
|  | ⭘ | ⭘ | ⭘ | ⭘ | ⭘ |
|  | I have never been irked when people expressed ideas very different from my own | | | | |
|  | ⭘ | ⭘ | ⭘ | ⭘ | ⭘ |
|  | I have never deliberately said something that hurt someone’s feelings | | | | |
|  | ⭘ | ⭘ | ⭘ | ⭘ | ⭘ |

**We thank you for your participation in this survey!**
